# Interventional sialendoscopy in parotidomegaly related to eating disorders

**DOI:** 10.1186/s40337-021-00378-9

**Published:** 2021-02-17

**Authors:** Giuseppe Colella, Giorgio Lo Giudice, Roberto De Luca, Antonio Troiano, Carmelo Lo Faro, Vincenzo Santillo, Gianpaolo Tartaro

**Affiliations:** 1 Multidisciplinary Department of Medical-Surgical and Dental Specialties, Oral and Maxillofacial Surgery Unit, University of Campania “Luigi Vanvitelli”, 80138 Naples, Italy; 2 Department of Neurosciences, Reproductive and Odontostomatological Sciences, Maxillofacial Surgery Unit, University of Naples “Federico II”, 80138 Naples, Italy

**Keywords:** Sialendoscopy, Sialoendoscopy, Parotidomegaly, Sialadenosis, Sialoadenosis, Sialadenitis, Sialoadenitis, Eating disorders, Anorexia, Bulimia

## Abstract

**Background:**

To evaluate the viability and efficacy of sialendoscopy for the management of parotidomegaly related to eating disorders, 6 patients suffering from eating disorders and recurring symptoms of glandular swelling were followed up at the Multidisciplinary Department of Medical-Surgical and Dental Specialties, Oral and Maxillofacial Surgery Unit, AOU University of Campania “Luigi Vanvitelli”. After the detection of the impaired gland through clinical and radiographical analysis, the diagnostic unit was introduced into the duct and was advanced in, reaching the ductal system. Plaques were washed out, any strictures were dilated both by hydrostatic pressure application and steroid solution injection directly in the fibrotic area.

**Results:**

Both glands resulted affected in 83% of patients. 11 parotid glands were explored and treated. Strictures were found in 2 glands (33%), sialectasis in 3 glands (50%), strictures and sialectasis together in 1 glands (17%). In 3 parotid glands (50%) Stenon’s duct was affected, in two (33%) only secondary ducts, in 1 (17%) both. We reached symptomatic improvement in 5 patients (83%), reporting the spherical volume of the parotid region and pain reduction.

**Conclusions:**

Our results demonstrate that sialendoscopy is a safe and effective therapeutic method to treat EDs salivary symptoms. Treating the underlining psychiatric pathology should be the primary goal in patient care to lower the possible recurrence rate and increase the successful outcome of this technique.

## Plain English summary

Patients affected by Eating Disorders such as Anorexia Nervosa and Bulimia Nervosa often follow compensatory behaviors to control their weight. Among all behaviors, if self-induced vomiting is protracted over time, oral health can be affected on both anatomical and biochemical level. Salivary gland swelling is a pathological alteration that these patients can manifest. The glands can become visible and sometimes painful, changing the facial profile. Sialendoscopy is a technique that offers a minimally invasive approach to non-neoplastic diseases, allowing the endoscopic visualization of the salivary glands and their ducts, offering a tool to treat ductal system pathologies. The aim of this research was then to assess the validity of this non-invasive technique to treat salivary symptoms related to Eating Disorders. The results showed a reduction of salivary gland volume and symptom relief in most patients, opening a new path to treat the consequences of such conditions.

## Background

Eating disorders (EDs) are psychological conditions based on self-misperception of body shape and weight, often leading to severe systemic conditions that require medical treatment. DSM-V lists different behavioral syndromes in the Feeding and Eating Disorders chapter: Pica, Rumination Disorder, Avoidant/Restrictive Food Intake Disorder, Anorexia Nervosa (AN), Bulimia Nervosa (BN), Binge-Eating Disorder and Unspecified Feeding or Eating Disorder [1]. AN clinical characteristics lead to two subtypes: Anorexia Nervosa Restricting type (AN-R) and Anorexia Nervosa Binge-eating/Purging type (AN-BP) whereas BN is distinguished by compensatory actions after binge eating. Patients may follow inappropriate compensatory behaviors in order to avoid guilt perceived after eating or weight gain, such as chewing and spitting, self-induced vomiting, fasting or intake restriction after food consumption, drug intake such as laxatives or diuretics, inappropriate use of enemas, and over-exercising. The self-induced vomiting behaviour exhibited by some patients sabotages the oral health status, affecting both hard and soft tissues. Clinically, the patients may show enamel erosion, tooth decay, periodontal diseases, mucositis, and glossitis. The salivary profile is also affected both biochemically and anatomically showing altered salivary flow, increased amylase levels, decreased pH and bicarbonate salivary levels, salivary gland swelling (mostly affecting the parotid gland), sialadenosis, and facial swelling coming from parotidomegaly [2]. Although the mechanisms underlining salivary gland swelling are still up to debate, this condition must be treated by the physician: the impact on the patients’ body image is significant, furthermore magnified by the social pressure to pursuit a symmetric, imperfection-free face, possibly worsening their dysmorphic disorder. Nahlieli and Baruchin introduced rigid salivary gland endoscopy in 1994 [3]. Since that time, sialendoscopy has been used for diagnostic and therapeutic purposes [4]. Sialendoscopy offers a minimally invasive approach to non-neoplastic diseases, allowing endoscopic intraluminal visualization and offering a tool to treat ductal system pathologies, reducing or eliminating the need for sialadenectomy thus avoiding the related surgical risks [5–8]. This paper aims to evaluate the reliability of sialendoscopy in the management of parotidomegaly related to eating disorders.

## Materials and methods

Six patients were referred to the Multidisciplinary Department of Medical-Surgical and Dental Specialties, Oral and Maxillofacial Surgery Unit, AOU University of Campania “Luigi Vanvitelli” and were recruited for this prospective study from November 2012 to December 2016. The inclusion criteria were: diagnosis of EDs, recurring symptoms of glandular swelling either with or without pain, enlarged volume of the parotid region. Exclusion criteria were: any previous sialendoscopic treatment and/or botulinum toxin treatment of the parotid, previous facial surgery.

Patients were diagnosed with EDs by our center according to DSM-V. ED related parotidomegaly and sialadenosis was diagnosed after clinical and radiological examination, and exclusion of drinking history, parotid gland infective diseases, sialolithiasis or tumors.. Morphological analysis was performed through ultrasound and MRI scans. The spherical volume of the parotid region was measured according to Metzger at baseline (T0) and 6 months follow-up (T1) as “V = 4/3 × π × (r1 × r2 × r3)” where r1, r2, and r3 are the semidiameters for gland height, width, and depth [9]. Signs and symptoms improvement were considered as the primary endpoint of this study and it was evaluated assessing salivary swelling and pain reduction compared to baseline.

After the detection of the impaired gland, local anesthesia with lidocaine 2% was achieved on the orifice region. Gradual dilatation of the duct orifice was then performed, using salivary probes of increased diameter from 0000 to 0 size and with a 0.5 mm lacrimal probe, reaching 1.3 mm diameter, matching the outer diameter of the sialendoscope diagnostic unit (Marchal Sialendoscope - Karl Storz). The larger scopes (1.6 mm diameter) were introduced as needed and a 5 mm papillotomy was performed in order to prevent false roads creation. The diagnostic unit was introduced into the duct and was advanced, until reaching the ductal system while carrying out continuous lavage with isotonic saline solution. Care is taken to avoid puncturing or lacerating the duct. The plaques were washed out; any strictures were dilated both by hydrostatic pressure application and steroid solution injection directly in the fibrotic area; mucous plugs and debris were removed with irrigation or forceps. At the end of the procedure, the ductal system was irrigated with a betamethasone solution (Bentelan 4 mg/2 ml – Alfasigma S.p.a., Milano, Italy) under direct vision while withdrawing the scope, in order to treat the inflammation of the ductal epithelium (sialodochitis) and to promote the dilatation of ductal strictures.

Sialendoscopy was considered successful when the entire ductal lumen and its branches were clear of any disease. An antibiotic prophylaxis treatment was performed with twice a day doses of Amoxicillin 875 mg and Clavulanic acid 125 mg on post-operative phase for one week. The patients were followed-up at 3 months, 6 months (T1), and 1 year.

## Results

**Fig. 1:**
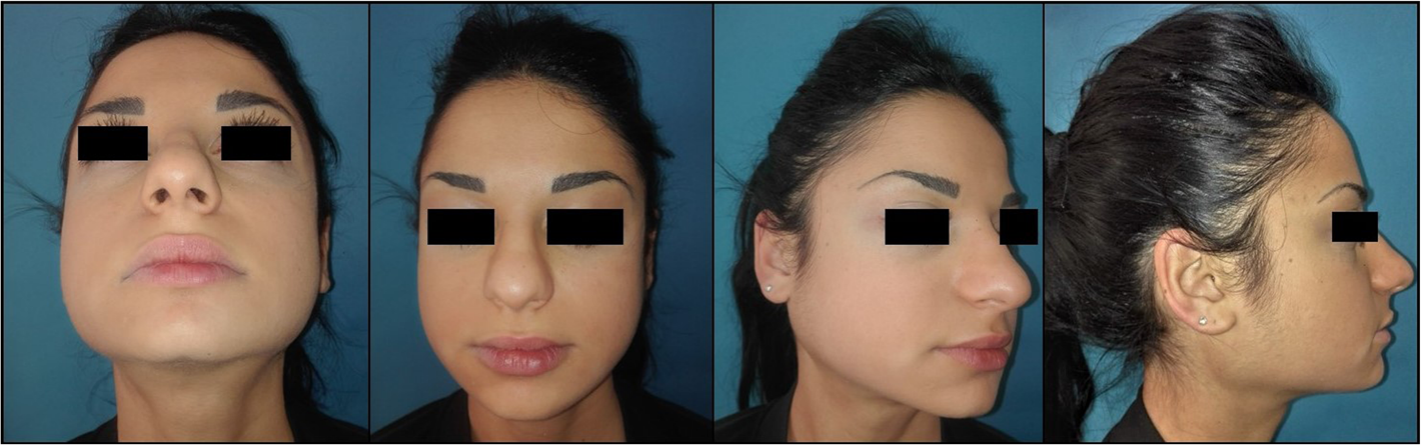
T0 external view examination

Our cohort included 6 female patients with a mean age of 22 years. 4 patients came to our attention with a diagnosis of AN-BP, 2 with a diagnosis of BN. In patients 1, 3 and 4, eating disorders familiarity was discovered during anamnesis. In patients 2 and 4, Generalized Anxiety Disorder and Major Depression treated with benzodiazepines were associated. Symptoms onset average from our medical examination was 12.6 months (Table 1, Fig. 1).

**Table 1:** Patients’ demographics. (AN-BP - *Anorexia Nervosa Binge eating/Purging type; BN - Bulimia Nervosa*)

Patient	Sex	Age	Symptoms Onset (months)	Eating Disorder	Eds Familiarity	Comorbidities	Ultrasound Findings
1	F	18	12	BN	yes	None	Hypoechogenic areas and punctate calcifications
2	F	23	6	AN-BP	no	Generalized Anxiety Disorder and Major Depression	Hypoechogenic areas
3	F	25	6	BN	yes	None	Hypoechogenic areas and reactive lymph nodes
4	F	27	18	AN-BP	yes	Generalized Anxiety Disorder and Major Depression	Hypoechogenic areas and punctate calcifications
5	F	17	4	AN-BP	no	Autoimmune Thyroiditis	Hypoechogenic areas
6	F	20	3	AN-BP	no	DM1, Fibromialgia	Hypoechogenic areas

**Fig. 2:**
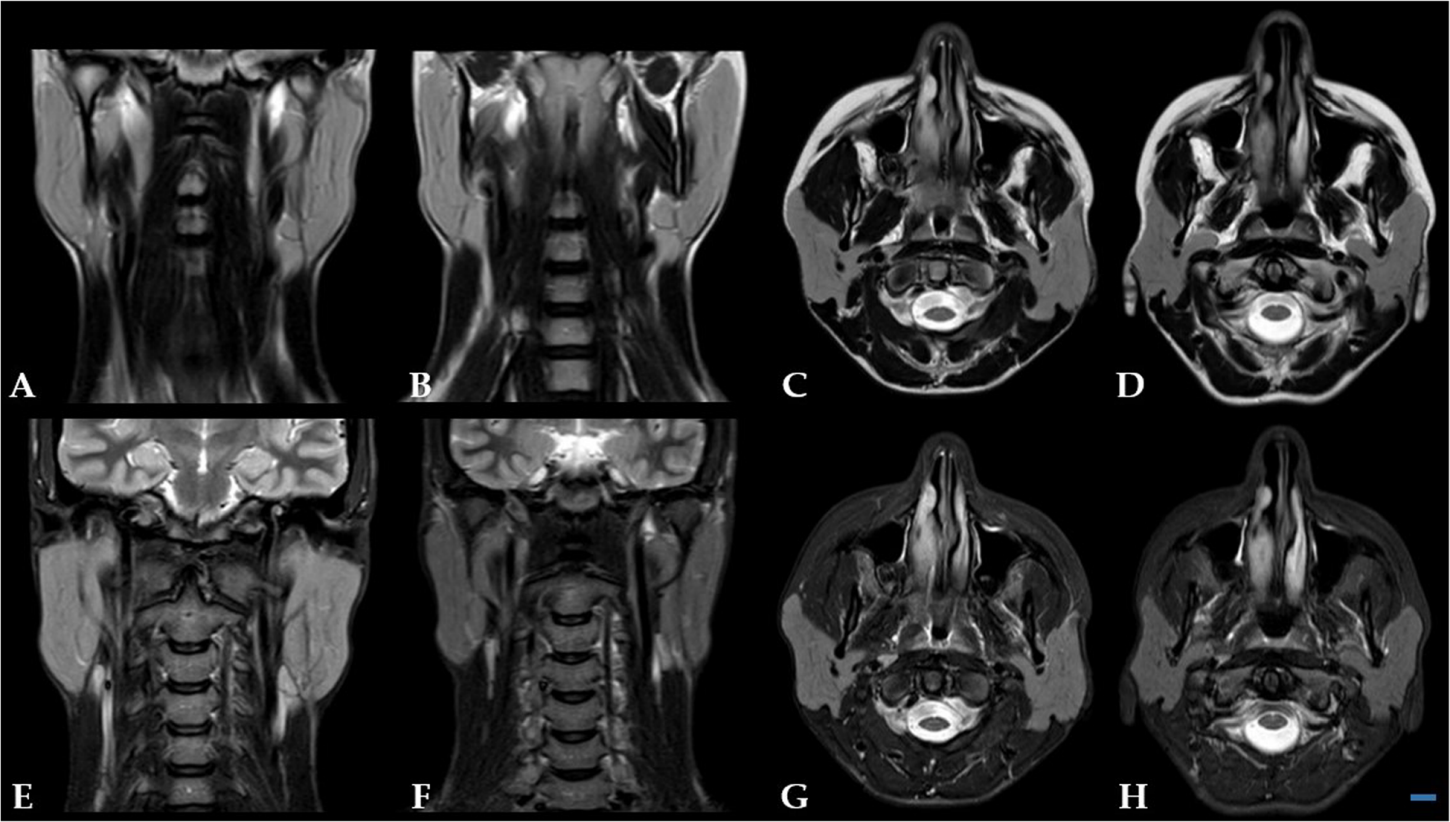
MRI assessment shows reduction of the parotid gland volume bilaterally. Coronal T2w at T0 (**a**) and T1 (**b**); axial T2w at T0 (**c**) and T1 (**d**); coronal STIR at T0 (**e**) and T1 (**f**); axial STIR at T0 (**g**) and T1 (H). BAR: 1 cm

Before performing this technique, all patients carried out analgesic and antibiotic drug therapy without any relief. All patients performed ultrasonography and MRI scans: in 3 patients the preoperative imaging evidenced hypoechogenic areas, in 2 patients hypoechogenic areas and punctate calcifications, in 1 patient hypoechogenic areas and reactive lymph nodes (Table 1, Fig. 2).

Both glands resulted affected in 5 out of 6 patients (83%). 11 parotid glands were explored and treated; in 5 patients the procedure was completed with no complications. However, in 1 case the procedure was discontinued at the initial stage due to pain intolerance and anxiety. The procedure was then performed again after 1 week (Table 2).

**Table 2:** Sialendoscopic features and outcomes. (*StD - Stenon’s duct; SeD - Secondary ducts*)

Patient	Gland	Findings	Intervention	Complications	Parotid region spherical volume (T0)	Parotid region spherical volume (T1)
1	L Parotid R Parotid	Sialectasis StD	Dilatation and removal	None	5 cm L 4,8 cm R	4.5 cm L 3.2 cm R
2	R Parotid	Stricture StD Stricture SeD	Dilatation and removal	None	4.5 cm	2 cm
3	L Parotid R Parotid	Stricture SeD	Dilatation and removal	None	2.9 cm L 4 cm R	1.2 cm L 3 cm R
4	L Parotid R Parotid	Stricture SeD Sialectasis SeD	Dilatation and removal	None	3.7 cm L 4.4 cm R	2.1 cm L 3.2 cm R
5	L Parotid R Parotid	Sialectasis StD	Dilatation and removal	None	6.2 cm L 6.5 cm R	2.5 cm L 1.8 cm R
6	L Parotid R Parotid	Sialectasis StD	Dilatation and removal	Failed 4 endoscopies	6.3 cm L 4.5 cm R	6.2 cm L 4.5 cm R

**Fig. 3:**
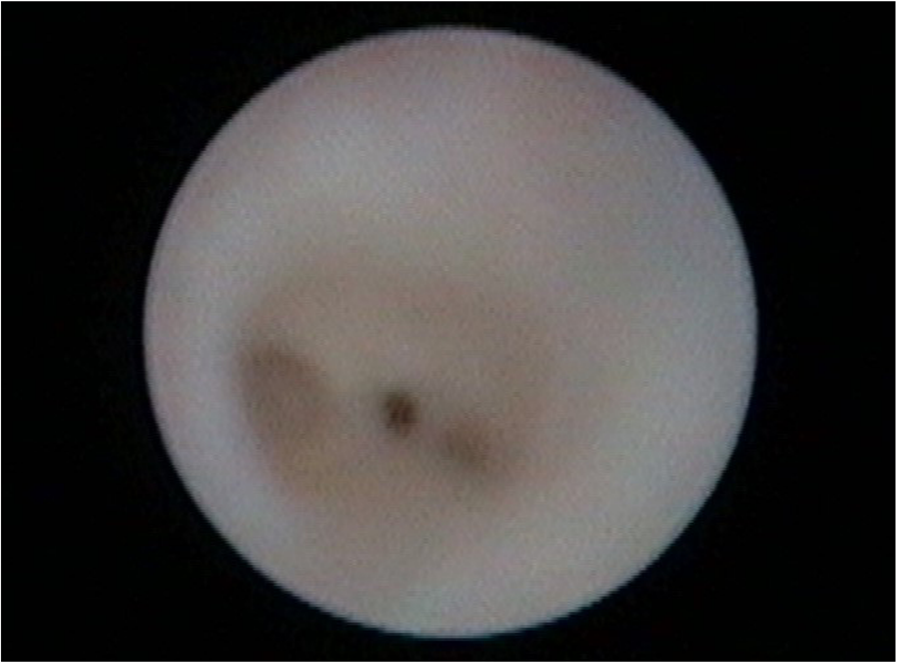
Sialendoscopic photogram of right parotid primary duct stricture

Strictures were found in 2 glands (33%), sialectasis in 3 glands (50%), strictures and sialectasis together in 1 gland (17%). In 3 parotid glands (50%) Stenon’s duct was affected, in 2 glands (33%) only secondary ducts, in 1 (17%) both (Fig. 3).

**Fig. 4:**
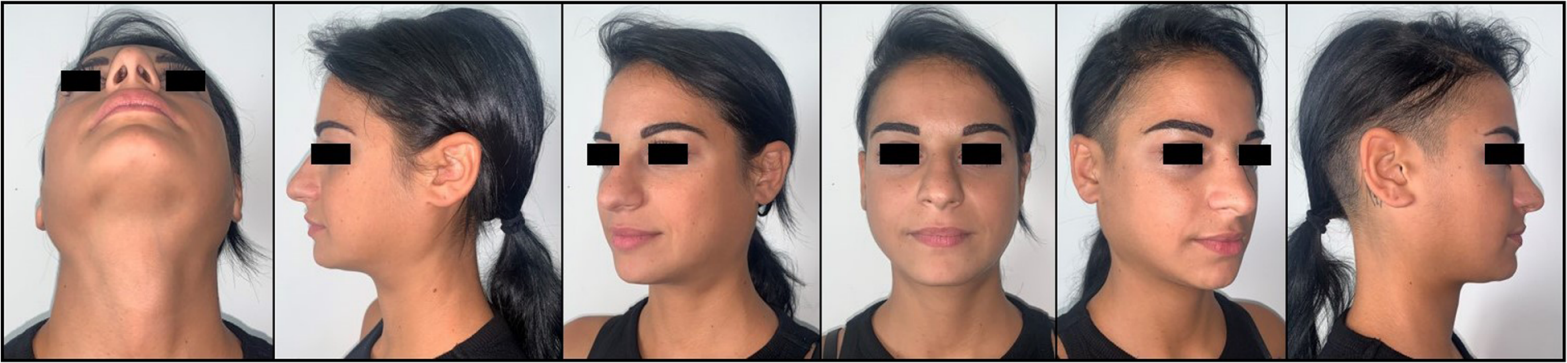
T1 external view examination

No diagnosis of sialolithiasis was made in our patients. No major complications such as nerve damage, hemorrhage, airway impairment, ductal perforation or kinks were observed. Symptomatic improvement was reached in 5 patients at T1 (83%). The patients showed spherical volume reduction of the parotid region and pain reduction at T1 (Table 2, Fig. 4).

One patient (patient 6) with bilateral parotidomegaly did not reach symptom relief nor spherical volume reduction after performing 4 sialendoscopies in succession at a time interval of 6 months. In this case, we observed an endoscopic pattern of sialectasis bilaterally. One patient (patient 5) did not show immediate improvement and they underwent a further sialendoscopy within a 3–5 months interval from their first treatment, obtaining success.

## Discussion

Studies found in literature describe non-inflammatory swelling of the salivary glands as a symptom underlining eating disorders [10, 11]. The enlarged area of the anatomical region of the parotid gland and, occasionally, of the submandibular gland, are described in patients with AN and BN, while other glands may not show any macroscopic alterations [12, 13]. Parotid swelling incidence has been estimated from 10 to 50% and may be either uni- or bilateral [14–17]. Some studies presume the etiology of this condition to be a combination of an abnormally low body mass index (BMI), nutritional deficiency, functional hypertrophy, and neurovegetative/hormonal dysregulation. Binge eating and purging behaviour by vomiting seems to be directly connected to the onset of the swelling, usually beginning 2–6 days after the trigger episode. This sign tends to fade in early stages if the behaviour is not protracted in time and is generally painless [18]. Histological examinations show changes within the parenchyma of the parotids, including hypertrophy of the cells and increased storage of adipose tissue [10, 14–16]. Hyposalivation is another typical feature reported in patients affected by both AN subtypes and BN, thus possibly concurring to the persistence of the glands inflammatory state. The genesis of this phenomenon could come from vomiting, starvation and antidepressant medications that have antisialagogue side effects. The appetite suppressant may also play a role in this pathological sign [2, 19]. Since this plethora of signs tend to subside after ED treatment and resolution, due to the inflammatory trigger removal, a step-by-step treatment approach should be followed: intensive pharmacological, sialendoscopic and then surgical approaches should be kept for refractory cases in long-history ED patients. Reports of effective sialogogue drug use such as pilocarpine, can be found in literature and could be used as primary treatment in such patients [20, 21].

While performing sialoendoscopy, phlogistic findings were detected. Strictures were founded in 2 glands (33%), sialectasis in 3 glands (50%), strictures and sialectasis together in 1 gland (17%). Each of these features, typical of chronic sialoadenitis, is widely described in literature and successfully treated with sialendoscopy [21, 22].

Sialendoscopy is a technique able to offer a minimally invasive and gland-preserving approach to non-neoplastic salivary glands diseases. In our work, we obtained encouraging results that justify the possibility of treating EDs salivary symptoms with a conservative procedure reaching symptomatic improvement in most of the patients treated. The morphological analysis showed spherical volume reduction of the parotid region, less incidence of salivary swelling and pain reduction (Table 2). Only 1 patient with bilateral parotidomegaly reached neither symptoms relief nor spherical volume reduction after performing 4 sialendoscopies in succession at a time interval of 6 months. Botulinum toxin injection of the salivary glands is used to treat sialorrhea, salivary fistulae, first bite syndrome, Frey syndrome and sialoadenitis [23]. Cosmetic off-label use has been reported in literature due to its parenchymal atrophy effects in order to reduce gland volume thus performing facial recontouring [24]. This treatment, reported to be possible in both percutaneous and intraductal infusion, could be used to treat those parotidomegaly cases refractory to the sialendoscopic therapy [25]. The surgical treatment (superficial or total parotidectomy) should be carefully pondered by the surgeon and used as a last resort, considering that a slight significant percentage of symptomatic pain is usually referred, the young mean age of the patients in question, and the related risks of such surgical procedures.

## Conclusions

Sialendoscopy is a versatile procedure, worthwhile to treat EDs salivary symptoms refractory to common therapy. Despite the small cohort of patients considered, our results are promising and should push the researchers to widen its application fields. Treating the underlining psychiatric pathology should be the primary goal in patient care, in order to lower the possible recurrence rate and increase the successful outcomes of this technique.

## Data Availability

Data are available upon reasonable request from the corresponding author.

## Abbreviations

AN: Anorexia Nervosa
AN-BP: Anorexia Nervosa Binge eating/Purging type
AN-R: Anorexia Nervosa Restricting type
BN: Bulimia Nervosa
DSM-V: Diagnostic and statistical manual of mental disorders - 5th edition
ED: Eating disorder
MRI: Magnetic Resonance Imaging

## References

[1] American Psychiatric Association (2013). Diagnostic and statistical manual of mental disorders. 5th ed.

[2] Frydrych AM, Davies GR, McDermott BM (2005). Eating disorders and oral health: a review of the literature. Aust Dent J.

[3] Nahlieli O, Baruchin AM (1997). Sialoendoscopy: three years' experience as a diagnostic and treatment modality. J Oral Maxillofac Surg.

[4] Capaccio P, Torretta S, Ottavian F, Sambataro G, Pignataro L (2007). Modern management of obstructive salivary diseases. Acta Otorhinolaryngol Ital.

[5] Strychowsky JE, Sommer DD, Gupta MK, Cohen N, Nahlieli O (2012). Sialendoscopy for the management of obstructive salivary gland disease: a systematic review and meta-analysis. Arch Otolaryngol Head Neck Surg..

[6] Shacham R, Puterman MB, Ohana N, Nahlieli O (2011). Endoscopic treatment of salivary glands affected by autoimmune diseases. J Oral Maxillofac Surg.

[7] Marchal F, Dulguerov P, Becker M, Barki G, Disant F, Lehmann W (2001). Specificity of parotid sialendoscopy. Laryngoscope..

[8] De Luca R, Trodella M, Vicidomini A, Colella G, Tartaro G (2015). Endoscopic management of salivary gland obstructive diseases in patients with Sjogren's syndrome. J Craniomaxillofac Surg.

[9] Metzger ED, Levine JM, McArdle CR, Wolfe BE, Jimerson DC (1999). Salivary gland enlargement and elevated serum amylase in bulimia nervosa. Biol Psychiatry.

[10] Bozzato A, Burger P, Zenk J, Uter W, Iro H (2008). Salivary gland biometry in female patients with eating disorders. Eur Arch Otorhinolaryngol.

[11] Walsh BT, Croft CB, Katz JL (1981). Anorexia nervosa and salivary gland enlargement. Int J Psychiatry Med.

[12] Buchanan JA, Fortune F (1994). Bilateral parotid enlargement as a presenting feature of bulimia nervosa in a post-adolescent male. Postgrad Med J.

[13] Het S, Vocks S, Wolf JM, Hammelstein P, Herpertz S, Wolf OT (2015). Blunted neuroendocrine stress reactivity in young women with eating disorders. J Psychosom Res.

[14] Coleman H, Altini M, Nayler S, Richards A (1998). Sialadenosis: a presenting sign in bulimia. Head Neck.

[15] Du Plessis DJ (1956). Parotid enlargement in malnutrition. S Afr Med J.

[16] Vavrina J, Muller W, Gebbers JO (1994). Enlargement of salivary glands in bulimia. J Laryngol Otol.

[17] Mandel L, Kaynar A (1992). Bulimia and parotid swelling: a review and case report. J Oral Maxillofac Surg.

[18] Brown S, Bonifazi DZ (1993). An overview of anorexia and bulimia nervosa, and the impact of eating disorders on the oral cavity. Compendium..

[19] Tschoppe P, Wolgin M, Pischon N (2010). Etiologic factors of hyposalivation and consequences for oral health. Quintessence Int.

[20] Mehler PS, Wallace JA (1993). Sialadenosis in bulimia. A new treatment. Arch Otolaryngol Head Neck Surg.

[21] Mignogna MD, Fedele S, Lo RL (2004). Anorexia/bulimia-related sialadenosis of palatal minor salivary glands. J Oral Pathol Med.

[22] Ziegler CM, Steveling H, Seubert M, Muhling J (2004). Endoscopy: a minimally invasive procedure for diagnosis and treatment of diseases of the salivary glands. Six years of practical experience. Br J Oral Maxillofac Surg.

[23] Gillespie MB, Intaphan J, Nguyen SA (2011). Endoscopic-assisted management of chronic sialadenitis. Head Neck..

[24] Jung GS, Cho IK, Sung HM (2019). Submandibular gland reduction using Botulinum toxin type a for a smooth jawline. Plast Reconstr Surg Glob Open.

[25] Schwalje AT, Hoffman HT (2019). Intraductal salivary gland infusion with Botulinum toxin. Laryngoscope Investig Otolaryngol.

